# Rethinking dog stress behaviours: contextual and physiological insights from the attachment framework

**DOI:** 10.1080/01652176.2026.2682284

**Published:** 2026-08-21

**Authors:** Giacomo Riggio, Carmen Borrelli, Durmus Atilgan, Giulia Russo, Silvana Diverio, Chiara Mariti

**Affiliations:** a Department of Veterinary Sciences, University of Pisa, Pisa, Italy; b Department of Physiology, Faculty of Veterinary Medicine, Ankara University, Ankara, Turkey; c Department of Veterinary Medicine, University of Perugia, Perugia, Italy

**Keywords:** Cortisol, dog-human relationship, oxytocin, strange situation, stress

## Abstract

While dogs’ behavioural indicators of psychological stress are widely used, their function as reliable stress markers remains debated. Using the Strange Situation Procedure (SSP) as a social-stress paradigm, we evaluated contextual correlates of dog behaviours to explore their validity as stress indicators. In 64 dogs, behaviours commonly used as stress markers were video-coded and analysed via GLMMs to investigate differences across test episodes and attachment styles (secure, anxious, avoidant), while salivary cortisol, oxytocin, and rectal temperature provided a physiological framework. Episode had significant effects on shaking (*p* < 0.05), lip-licking (*p* < 0.001), paw-lifting (*p* < 0.001), whining (*p* < 0.001), head-turning (*p* < 0.001), sniffing the door (*p* < 0.001), and locomotion (*p* < 0.001), while attachment style significantly affected head-turning only (*p* = 0.004). Our findings suggest that the observed behaviours were not expressed linearly in relation to expected stress levels across the SSP episodes. This lack of linearity, combined with the divergent physiological correlates found across attachment groups—for instance, oxytocin correlated positively with lip-licking in securely attached dogs (*p* < 0.05), but negatively in anxious ones (*p* < 0.05) –, suggests that these behaviours do not function as simple stress indicators. Rather, their meaning appears to be contingent upon the individual's internal state and the socio-environmental context. We discuss our results in light of an alternative semiotic interpretative framework.

## Introduction

The number of owned dogs has increased significantly in recent decades, to the point that they have become an integral part of human society and, in some cultures, stable members of the closest family unit (Gillet and Kubinyi 2025). Nonetheless, our understanding of dogs’ behavioural expressions of psychological stress remains considerably limited (de Winkel et al. 2024). This gap has important welfare implications, as misinterpretation of behaviours occurring in potentially stressful contexts may lead to inappropriate management and interactions, ultimately compromising both canine and human safety and wellbeing. To date, virtually every research protocol that assesses stress in dogs through behavioural observation relies on behaviours that have historically been grouped under the broad label of ‘stress behaviours’ or, sometimes ‘displacement behaviours’ - normal activities exhibited in contexts where they appear irrelevant, arising from internal motivational conflict or when goal-directed behaviour is blocked by internal or external factors (e.g. frustration) (Troisi 2002; Pastore et al. 2011)—or, ‘appeasement signals’—postures or actions displayed by the dog to prevent or de-escalate conflict during agonistic encounters (Rugaas 2006; Firnkes et al. 2017; Mariti et al. 2017), and have often been used as direct proxies for stress. Behaviours commonly included under these umbrella definitions are lip-licking, shaking, paw-lifting, head-turning, avert gazing, self-scratching, stretching, yawning, sniffing, locomotor activity, vocalisations (Hetts et al. 1992; Beerda et al. 1997; Beerda et al. 1999, 2000; Schilder and Van Der Borg 2004; Rooney et al. 2007; Frank et al. 2007; Haverbeke et al. 2008; Doring et al. 2009; Mariti et al. 2012; Kuhne et al. 2014; Deldalle and Gaunet 2014; Kiddie and Collins 2014; Grigg et al. 2017; Corsetti et al. 2019; Townsend and Gee 2021; Dini et al. 2025). Over time, both the validity—i.e. their direct relationship with stress—and the universal applicability—i.e. their consistent function across contexts—of some of these behaviours as stress indicators in dogs have been called into question (Part et al. 2014; Albuquerque et al. 2018; Pedretti et al. 2023).

For instance, lip licking has been observed during interactions of both putative positive and negative valence in both intraspecific and interspecific contexts (Kuhne 2016; Caeiro et al. 2017; Bremhorst et al. 2019; Bremhorst et al. 2022; Pedretti et al. 2022; Pedretti et al. 2023), leading some authors to suggest that this behaviour may indicate more general state of elevated arousal (Rehn and Keeling 2011). Body shaking has been proposed as a play soliciting behaviour (Bekoff 1974), as an expression of relief after a stressful event (Beerda et al. 2000), and more recently, as a marker of behavioural transition, not necessarily related to or caused by stress (Bryce et al. 2024). Paw-lifting has been interpreted as a sign of motivational ambivalence (Horváth et al. 2007), uncertainty, pointing and a meta-signal preceding both playful and agonistic encounters (Beerda et al. 1997; Handelman 2008; Bekoff 2023). Attributing these behaviours exclusively to stress, or to specific motivations or intentions, as when labels such as ‘displacement’ or ‘appeasement’ are used, risks limiting their interpretation to a single cause which, although potentially present in certain circumstances, fails to explain the universality of the information these behaviours are intended to convey. These conceptual limitations may be addressed by adopting an interpretative framework that accounts for the functional flexibility of animal signals.

According to John Smith's Message and Meaning model (Smith 1965, 1997), signals consist of two distinct components: the message and the meaning. The message represents the information inherently encoded in the signal and remains constant across contexts; it refers to the sender’s behavioural referents, providing information about the likelihood of subsequent actions (e.g. a propensity for behavioural transition or social engagement). Conversely, the meaning is the receiver’s interpretation of the message, dynamically generated by integrating the latter with the specific context and prior experience, and may encompass information about the sender’s internal state (e.g. motivation, emotion, intention) (Smith 1965). This distinction allows us to understand why a single behaviour may be observed in diverse scenarios: while the underlying message remains universal, the resulting meaning— e.g. internal state associated with tail wagging during conflict versus reunion—is context-dependent.

With this study, we aimed to test the validity of the traditional categorical approach to dog behaviour, which interprets specific behaviours as indicators of stress, against a more flexible semiotic model, namely the Message and Meaning model. This model allows stable and context-dependent components of a signal to be considered separately and subsequently integrated to interpret the complexity of canine communication. For this purpose, we used the Strange Situation Procedure (SSP) as a contextual framework.

The SSP was originally developed to assess the quality of the attachment bond between a child and their mother (Ainsworth and Wittig 1969; Ainsworth and Bell 1970), and has since been widely used to investigate attachment behaviour patterns in dogs toward their caregivers (Topál et al. 1998, 2005; Schöberl et al. 2016; Solomon et al. 2019; Riggio et al. 2021), namely secure, insecure anxious/ambivalent, insecure avoidant, and disorganised. Although dogs’ behavioural and physiological responses to the SSP may vary depending on their attachment style (Schöberl et al. 2016; Ryan et al. 2019; Riggio et al. 2022), several studies have confirmed that the procedure elicits a stress response, which is necessary to activate the attachment system (Palestrini et al. 2005; Fallani et al. 2007; Ainsworth et al. 2015; Riggio et al. 2021). Due to its episode-based structure—where specific episodes are designed to induce stress (e.g. separation from the owner in the presence [episode 3] or absence [episode 5] of an unfamiliar person) and others to alleviate it (e.g. reunion with the caregiver [episodes 4 and 7], or to a lesser extent with the stranger [episode 6])—the SSP may provide a suitable experimental framework for investigating the contextual features of behaviours expressed by dogs during challenging situations.

In order to provide a physiological context for the observed behaviours, we measured variables associated with the activation of three physiological systems, namely, the sympathetic nervous system, the hypothalamic–pituitary–adrenal (HPA) axis, and the oxytocinergic system, all of which are implicated in social responses such as those elicited by the SSP. Specifically, body temperature is commonly used as an indicator of sympathetic nervous system (SNS) activation and is therefore considered a physiological parameter of the acute stress response in dogs (Travain et al. 2015; Bragg et al. 2015; Csoltova et al. 2017; King et al. 2022). Cortisol is a glucocorticoid hormone primarily secreted when the organism needs to adjust its metabolism to meet anticipated novel demands (Sapolsky et al. 2000). For this reason, it is often used as a complementary physiological parameter in the assessment of dog stress responses (Marliani et al. 2024). Preliminary findings suggest that dogs with different attachment patterns may exhibit varying levels of cortisol reactivity to stress-inducing events (Schöberl et al. 2016; Riggio et al. 2022). Oxytocin appears to be involved in the context-dependent modulation of social behaviours, possibly by enhancing the salience of social stimuli (Averbeck 2010; Shamay-Tsoory and Abu-Akel 2016). In the context of the dog–owner relationship, findings on the association between oxytocin concentrations and social behaviours have been inconsistent. While some studies report a positive association (Handlin et al. 2011; Nagasawa et al. 2015, Nagasawa and Tomori, 2024), others have found no association (Marshall-Pescini et al. 2019), or even a negative one (Hernádi et al. 2015; Thielke et al. 2017; Powell et al. 2019; Dzik et al. 2021). Furthermore, to the best of our knowledge, oxytocin levels have never been directly correlated with the behavioural patterns described above in dogs.

Our sole a priori hypothesis was that the frequency and duration of behaviours commonly labelled as stress indicators would vary across episodes of the SSP, not linearly to expected stress levels, but in relation to the specific social and environmental context of each episode. Given the conflicting literature on the biological function of these behaviours, we did not predict the direction of contextual influences (e.g. owner presence/absence, stranger presence/absence, attachment style) on individual behaviours. Instead, we explored the applicability of an alternative interpretative framework, to explain the observed behavioural patterns, taking into account the social and environmental characteristics of each episode. Due to both this conceptual approach and experimental design constraints (e.g. collection of biological samples only pre- and post-test, but not during individual episodes), analyses of associations between physiological and behavioural data were treated as exploratory, and aimed at generating hypotheses about the context-related meaning of behaviours for future testing.

## Materials and methods

Ethical approval for this study was obtained from the Committee on Bioethics of the University of Pisa, Italy (review no. 29/2021) for human participation and the Animal Welfare Review Board of the University of Pisa (review no. 31/2021) for animal participation. The owners’ informed consent and authorisation to video record and use the data for research purposes were obtained before each test.

### Subjects

The initial sample consisted of 66 dogs. However, two dogs, both classified as having a disorganised attachment, were excluded, as they were the only ones in that category. Table 1 summarises the dog’s demographic information for the final 64 subjects.

**Table 1.** Summary of the dogs’ demographic information. For the ‘Breed’ variable only the 5 most represent breeds are reported.

Variable	Number (%)	Mean (SD)	Min-Max
Age		6.20 (3.29)	1−14
Sex Male Female	24 (37.5) 40 (62.5)		
Neutering status Yes No	33 (51.6) 31 (48.4)		
Size Mini Small Medium Large Giant	2 (3.1) 12 (18.8) 27 (42.2) 23 (35.9) 0 (0.0)		
Breed Mix-breed Labrador Retriever Australian Shepherd Golden Retriever Border Collie	21 (32.8) 6 (9.4) 4 (6.3) 3 (4.7) 3 (4.7)		
Origin Amateur Breeder Pro Breeder Shelter/Rescue Born in the house Found on the street Shop	20 (31.3) 19 (29.7) 14 (21.9) 7 (10.9) 3 (4.7) 1 (1.6)		
Age at adoption 0-2 months 3-6 months 6 months-1year 1-2 years 2-8 years	27 (42.2) 26 (40.6) 2 (3.1) 6 (9.4) 3 (4.7)		

SD = Standard deviation.

### Experimental setting

**Figure 1.**
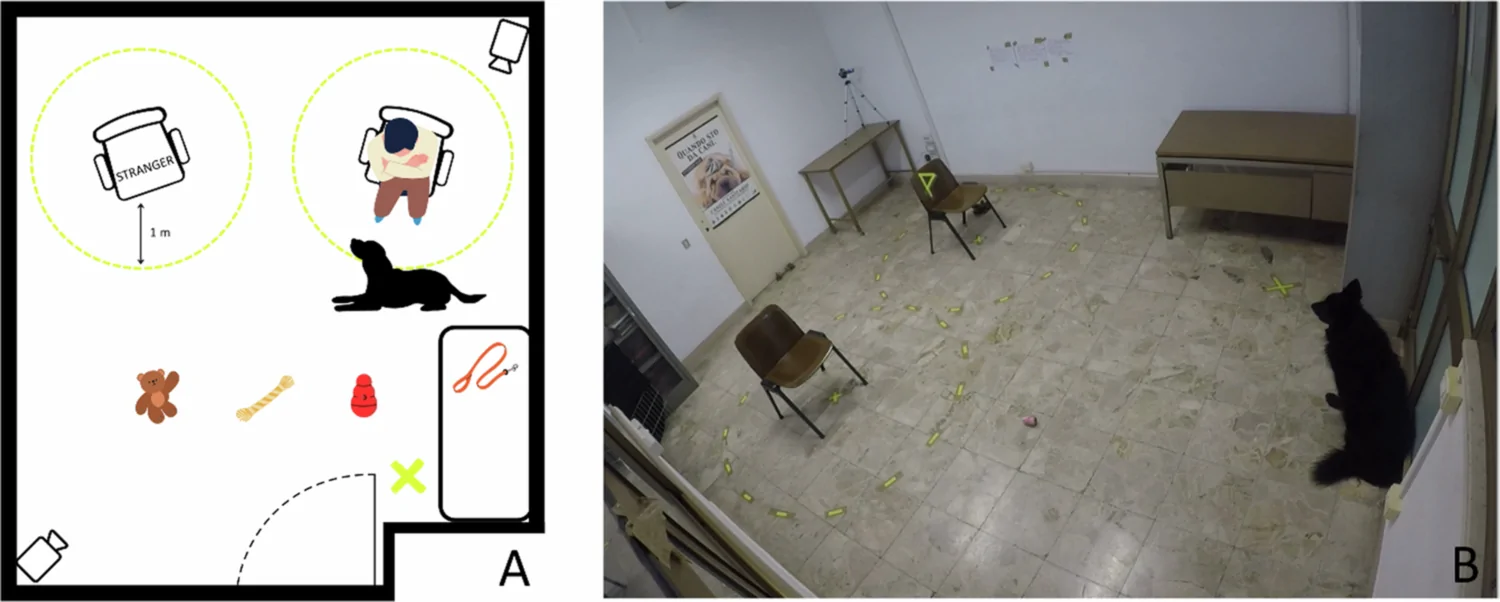
Experimental setting: A) schematic map of the experimental room; B) still image from the main video camera (episode 5). Yellow tape on the floor was used both to guide the owners during the procedure and to help researchers calculate distances during behavioural observations.

The experimental setting was a bare room inside the Department of Veterinary Sciences of the University of Pisa, Italy. The room, which measured approximately 4.50 m × 4.30 m, was prepared according to the description of the original SSP setting (Salter Ainsworth 1985), as well as the modified setting later used to specifically test dogs (Palmer and Custance 2008; Mariti et al. 2013). The room was equipped with two chairs, one for the owner and one for the stranger and three different toys - a rope, a stuffed animal, and an empty Kong^®^ (Golden, CO, USA) - placed on the floor in the middle of the room. In one corner of the room there was a table that the owners could use to drop their dog’s leash and harness. The room could be accessed or exited through a single door. Finally, two video cameras were placed at the two opposite corners of the room in order to record the whole test (Figure 1). None of the dogs had ever entered the experimental room before the test started. The tests were conducted on weekdays from 11:00 a.m. to 5:00 p.m., between August 2021 and July 2022.

### Experimental protocol

The dog–owner dyads underwent a Strange Situation Procedure (SSP), a 19-minute protocol conducted in a controlled room setting and designed to assess dogs’ attachment to their owner. The SSP consists of seven episodes: (1) the dog with the owner only, (2) the dog with the owner and a stranger, (3) the dog with the stranger only, (4) the dog with the owner only, (5) the dog alone, (6) the dog with the stranger only, and (7) the dog with the owner only again. Detailed descriptions of the SSP are available in Riggio et al. (2022), who used the same protocol as in the present study. Saliva samples were collected from the dogs to measure their salivary cortisol concentrations immediately before and after the end of the SSP. The detailed procedures for saliva collection and analysis are described in the ‘Physiological data collection’ section.

### Behavioural data collection

The videos were analysed manually by one researcher with the Behavioural Observation Research Interactive Software (BORIS) V. 8. 25. 4 (Friard and Gamba 2016) to assess the behaviours of the dogs during the SSP. The ethogram used in this study comprised the following behaviours: shaking, lip-licking, paw-lifting, self-scratching/grooming, yawning, whining/yelping, barking, head-turning, stretching, escape attempts, locomotion, sniffing the door. A detailed description of all recorded behaviours along with the corresponding bibliographic reference is reported in Table 2. Behaviours analysed based on frequency were coded according to the following rule: if the dog performs the behaviour (e.g. barking, whining, scratching the door, etc.) continuously for more than 3 seconds, it was counted as an additional event.

**Table 2.** Ethogram of the stress behaviours used in the current study.

Behaviour	Measure	Episode	Description	References
Shaking	F	All	Move, shake the body with energy (not trembling)	Kuhne et al. (2014), Glenk et al. (2014), Palestrini et al. (2017), McCullough et al. (2018), Grigg et al. (2021), de Winkel et al. (2024)
Lip licking	F	All	Sticking the tongue out, licking the nose or the lips	Kuhne et al. (2014), Palestrini et al. (2017)
Paw-lifting	F	All	Lifting one of the forepaws	Glenk et al. (2014), Stellato et al. (2017), Jeong et al. (2020)
Self-scratching	F	All	Behaviours directed towards the subject's own body, like self-scratching, licking and nibbling	Kuhne et al. (2014), Palestrini et al. (2017), McCullough et al. (2018)
Yawning	F	All	Opening the jaws wide without vocalising	Kuhne et al. (2014), Palestrini et al. (2017), Jeong et al. (2020)
Whining	F	All	Cyclic high-pitched vocalisation, mouth often closed	McCullough et al. (2018), Jeong et al. (2020)
Barking	F	All	Low frequency and short-lasting vocalisation	McCullough et al. (2018), Jeong et al. (2020)
Head turning	F	All, except ep. 5*	Turning the head either to the side and back, or holding the head to one side after visual engagement with the person (either the person directly looking at the dog or the dog looking at the person)	Kuhne et al. (2014)
Stretching	F	All	Extending the forelimbs forward, placing them flat on the ground, while the hindquarters usually remain more elevated	Kuhne et al. (2014), Somppi et al. (2022)
Escape attempts	F	All	Scratching the bottom of the door or window (if present), jumping on the door to pull the handle	McCullough et al. (2018)
Sniffing the door	F	All	Standing or lying next to the door and sniffing usually the bottom chink of the door to inhale air from outside	Included by the authors for the purpose of the current study
Locomotion	D	All	Non-goal-directed movement around the room, characterised by continuous walking, pacing, or repeated trajectories, without engaging in targeted olfactory investigation, visual examination, or manipulation of the environment.	Palestrini et al. (2017)

F: Frequency (number of occurrences), D: Duration (seconds), * Head turning was not recorded in ep. 5 as the dog was alone in the room.

### Attachment classification

The behaviour of all dogs during the SSP was videorecorded and qualitatively assessed to classify each dog according to their attachment style towards the owner. The classification was based on the attachment style descriptions provided by Riggio et al. (Riggio et al. 2021). Dogs could be classified as secure, insecure anxious/ambivalent (hereafter, anxious), insecure avoidant (hereafter, avoidant) or disorganised. Table S1 reports the behavioural descriptions of the four attachment styles used to classify the dogs in the current study. Initially, two researchers experienced in dog attachment classification independently classified the experimental subjects. Inter-observer agreement was calculated on a preliminary sample of 27 dogs. The classification matched in 81.5% of cases (22/27), and Cohen’s Kappa was 0.63, indicating substantial agreement (McHugh 2012). In cases of initial disagreement (5/27), the dog’s attachment style was re-evaluated by both researchers following a thorough discussion. Ultimately, consensus was reached for all cases (27/27).

### Physiological data collection

Saliva samples were collected immediately before (T0) and after (T1) the SSP with Salivette® (Sarstedt, Rommelsdorft, Germany) swabs. Samples at T0 were collected in an indoor waiting area outside the test room. Owners were instructed to insert the swab gently under the tongue and inside the cheek pouches of the dog for 90 seconds, according to the methodology described in previous studies (Schöberl et al. 2016, 2017). A researcher supervised the whole process.

Samples at T1 were collected in the test room after the end of test. The procedure was the same as in T0 except that, this time, sampling was performed by the researcher, while the owner was asked to help holding the dog in position, in order to collect additional data on their interactive behaviour towards the dog (not presented in this study). The timing for saliva sampling at T1 was chosen in accordance with data reported in previous studies on the determination of dog salivary cortisol and oxytocin concentrations after a stressful event (Mongillo et al. 2013; Carlone et al. 2018; Ogi et al. 2020). Specifically, we wanted to perform the sampling procedure 15 minutes after the beginning of episode 5 of the SSP (separation from the owner). Given that episode 5 lasted 2 minutes and episodes 6 and 7 lasted 3 minutes each, sampling was performed 7 minutes after the conclusion of the SSP. All samples were immediately centrifuged and stocked at −20 °C in the Etovet laboratory of the Department of Veterinary Sciences-University of Pisa (Italy), until they were analysed for cortisol quantification with the Salimetrics Cortisol Enzyme Immunoassay Kit® (Salimetrics, Segrate, Italy).

### Statistical analysis

RStudio version 4.4.3 was used to conduct all statistical analyses. Shapiro-Wilk test was performed to assess the distribution of the data. Since the data was not normally distributed, we ran a generalised linear mixed model (GLMM) with repeated measures using the ‘glmmTMB’ function for each behaviour. ‘Individual dogs’ were included as random effects, whereas the following were treated as fixed effects:

– Episode: Episode 1 to Episode 7 for behavioural variables.
– Time: T0 vs T1 for cortisol and oxytocin.
– Attachment style: secure, avoidant and anxious.

We used the ‘DhARMA’ package for model diagnostics, including quantile-quantile (QQ) plots of residuals and residuals vs. predicted values, to guide the selection of the appropriate model family and zero-inflation term. We employed a stepwise approach to select the most appropriate error distribution for each behavioural variable. We initially fitted models using a Poisson distribution, the standard baseline for count data. However, for some behaviours diagnostic tests revealed significant overdispersion. To account for this, we transitioned to a Negative Binomial distribution, which incorporates a dispersion parameter to model the excess variance. Model selection was confirmed by comparing Akaike Information Criterion (AIC) values across Poisson and Negative Binomial distributions, ensuring the most parsimonious fit for each behavioural variable. No significant zero-inflation was detected in any of the fitted models. The Negative Binomial distribution appeared to be the best fit for lip-licking, paw-lifting, whining, head-turning, sniffing the door, and barking; whereas the Poisson distribution was the most appropriate for shaking. Stretching, self-scratching, yawning, and escape attempts were excluded from inferential analyses due to their low number of occurrences (<10%), which precluded reliable statistical modelling. Post-hoc analysis was performed when main effects and/or interactions were significant. The ‘emmeans’ function was used to perform pairwise comparisons of significant main effects, while the ‘contrast’ function was implemented to conduct planned comparisons of significant interaction effects—specifically, comparisons of different episodes within the same attachment style and comparisons of the same episode between different attachment styles. For all multiple comparisons, statistical significance was adjusted with Tukey correction. Results are reported on the log scale. Additionally, possible correlations relating dogs’ *Δ* salivary cortisol (T1-T0) concentrations, *Δ* salivary oxytocin (T1-T0) concentrations, rectal temperature to the total sum of each significant behavioural variable were examined within each attachment style. For all correlations, Spearman’s coefficient for non-parametric data was used. Given the exploratory nature of these analyses, *p*-values were not corrected for multiple comparisons in order to preserve sensitivity to potentially meaningful associations and reduce the risk of Type II errors. This approach was adopted to avoid overlooking patterns that may warrant further investigation, while acknowledging the increased risk of Type I error (Rubin 2017; García-Pérez 2023). For all tests, *p*-value significance was set at *p* < 0.05.

## Results

### Classification of the dogs’ attachment styles

Based on the four-style classification scheme previously described, 43 (65.2%) dogs were classified as secure, 11 (16.6%) as avoidant, 10 (15.2%) as anxious and 2 (3.0%) as disorganised. The two disorganised dogs were excluded from further analysis because of their limited sample size.

### Descriptive results for behavioural and physiological variables

Descriptive statistics are presented in Tables 3 and 4 for behavioural and physiological variables, respectively.

**Table 3.** Descriptive statistics for dog behaviours recorded during the SSP.

Behaviour	Mean ± SE						
	Ep. 1	Ep. 2	Ep. 3	Ep. 4	Ep. 5	Ep. 6	Ep. 7
Shaking	0.08 **±** 0.03	0.11 **±** 0.04	0.00 **±** 0.00	0.41 **±** 0.10	0.00 **±** 0.00	0.06 **±** 0.05	0.52 **±** 0.12
Lip-licking	0.22 **±** 0.08	1.22 **±** 0.24	1.31 **±** 0.28	2.03 **±** 0.24	0.80 **±** 0.20	1.78 **±** 0.30	1.64 **±** 0.20
Paw-lifting	0.28 **±** 0.13	2.00 **±** 0.46	0.75 **±** 0.19	2.81 **±** 0.53	0.44 **±** 0.11	1.84 **±** 0.37	2.31 **±** 0.46
Self-scratching	0.08 **±** 0.05	0.03 **±** 0.02	0.02 **±** 0.02	0.05 **±** 0.03	0.02 v 0.02	0.17 **±** 0.17	0.09 **±** 0.04
Yawning	0.00 **±** 0.00	0.19 **±** 0.05	0.08 **±** 0.04	0.16 **±** 0.06	0.03 **±** 0.02	0.09 **±** 0.04	0.14 **±** 0.04
Whining	0.19 **±** 0.14	0.16 **±** 0.08	4.42 **±** 0.99	0.97 **±** 0.33	3.89 **±** 0.72	4.12 **±** 0.99	0.95 **±** 0.28
Barking	0.00 **±** 0.00	0.16 **±** 0.08	0.95 **±** 0.48	0.53 **±** 0.26	1.62 **±** 0.50	0.78 **±** 0.41	0.23 **±** 0.11
Head-turning	1.84 **±** 0.25	4.98 **±** 0.46	3.42 **±** 0.40	3.80 **±** 0.30		5.20 **±** 0.50	3.34 **±** 0.36
Stretching	0.02 **±** 0.02	0.02 **±** 0.02	0.00 **±** 0.00	0.03 **±** 0.02	0.06 **±** 0.04	0.00 **±** 0.00	0.03 **±** 0.02
Escape attempts	0.00 **±** 0.00	0.00 **±** 0.00	0.45 **±** 0.20	0.03 **±** 0.03	0.76 **±** 0.25	0.28 **±** 0.13	0.02 **±** 0.02
Sniffing the door	0.42 **±** 0.07	0.25 **±** 0.07	2.23 **±** 0.28	0.38 **±** 0.10	1.36 **±** 0.20	1.13 **±** 0.23	0.16 **±** 0.05
Locomotion	1.08 **±** 0.53	7.50 **±** 1.61	18.16 **±** 2.68	5.31 **±** 0.83	9.59 **±** 1.92	10.30 **±** 1.80	4.80 **±** 0.95

All behaviours are reported as occurrences except Locomotion which is reported as duration (seconds). Head-turning was not recorded in Ep. 5.

**Table 4.** Descriptive statistics for the dog physiological variables measured before and after the SSP.

Variable	Mean ± SE	
	T0	T1
Cortisol (ng/ml)	0.179 ± 0.012	0.217 ± 0.014
Oxytocin (pg/ml)	375.092 ± 26.074	420.802 ± 33.349
Rectal temperature (°C)		38.65 ± 0.05

Rectal temperature was measured at T1 only.

### Differences in behaviours between attachment styles across episodes

**Figure 2.**
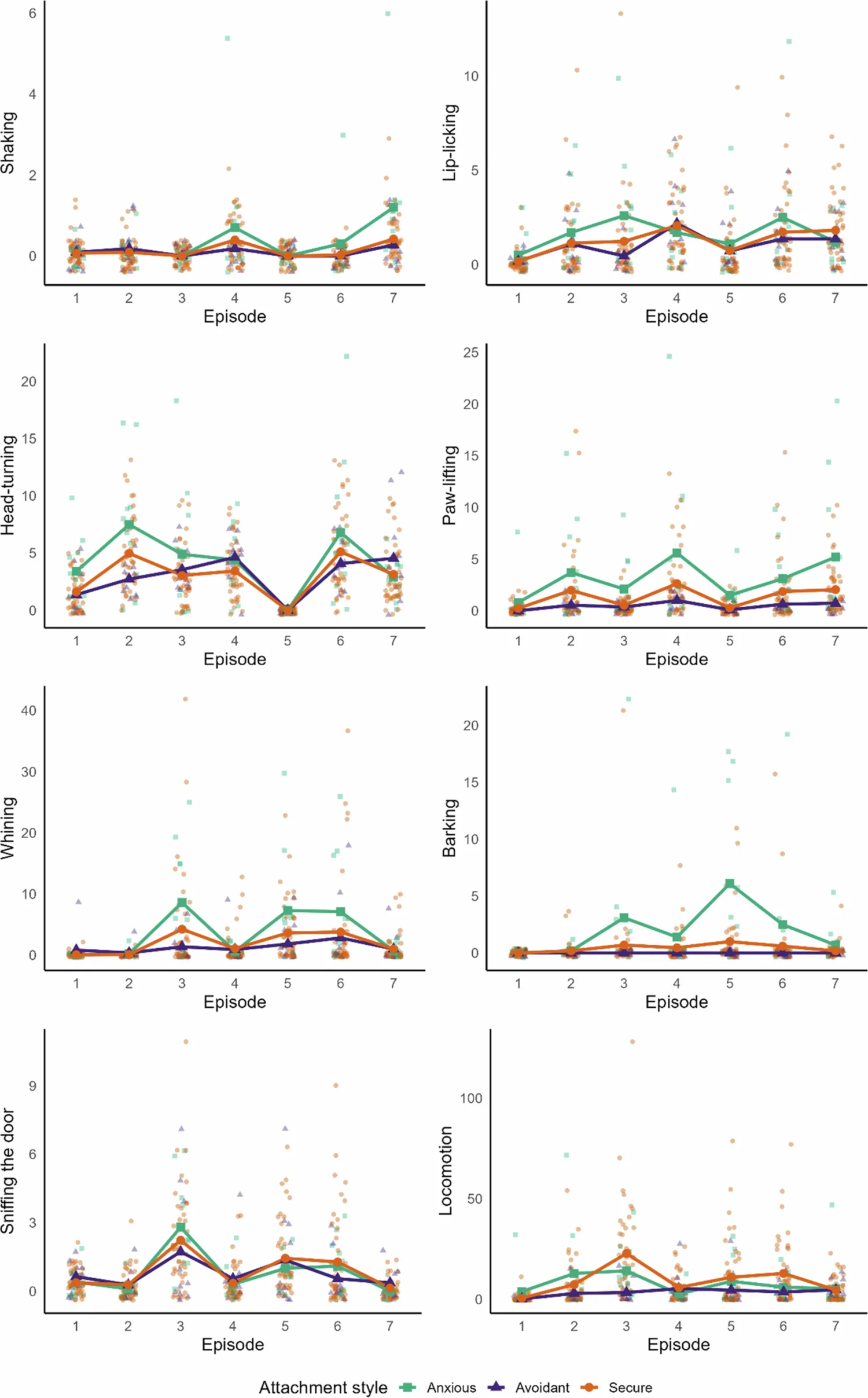
Expression of behaviours across episodes. All behaviours were recorded as frequency (occurrences), except for locomotion that was recorded as duration (seconds).

A significant main effect of ‘Episode’ emerged for several behaviours, including shaking (χ²(6) = 13.21, *p* = 0.039), lip-licking (χ²(6) = 49.25, *p* < 0.001), paw-lifting (χ²(6) = 36.53, *p* < 0.001), whining (χ²(6) = 110.75, *p* < 0.001), head-turning (χ²(6) = 53.65, *p* < 0.001), sniffing the door (χ²(6) = 67.46, *p* < 0.001), and locomotion (χ²(6) = 50.89, *p* < 0.001). For head-turning, there was also a significant interaction between ‘Episode’ and ‘Attachment Style’ (χ²(12) = 28.57, *p* = 0.004). No main effect of ‘Attachment Style’ was found for any behaviour. Changes in behavioural expression across episodes are illustrated in Figure 2, while significant pairwise comparisons are reported in Table 5.

**Table 5.** Statistically significant pairwise comparisons for each behaviour.

Behaviour	Pairwise	Estimate	SE	Z ratio	*P* value
Shaking	Ep 1 - Ep 7	−1.792	0.560	−3.209	0.023
	Ep 2 - Ep 7	−1.465	0.500	−2.948	0.050
Lip-licking	Ep 1 - Ep 2	−1.661	0.359	−4.631	<0.001
	Ep 1 - Ep 3	−1.534	0.373	−4.110	0.001
	Ep 1 - Ep 4	−2.172	0.352	−6.173	<0.001
	Ep 1 - Ep 5	−1.269	0.372	−3.409	0.012
	Ep 1 - Ep 6	−2.009	0.352	−5.701	<0.001
	Ep 1 - Ep 7	−1.818	0.359	−5.065	<0.001
	Ep 4 - Ep 5	0.902	0.238	3.784	0.003
	Ep 5 - Ep 6	−0.740	0.240	−3.087	0.033
Paw-lifting	Ep 2 - Ep 5	1.495	0.420	3.557	0.007
	Ep 3 - Ep 4	−1.211	0.276	−4.384	<0.001
	Ep 3 - Ep 7	−0.971	0.284	−3.419	0.011
	Ep 4 - Ep 5	1.943	0.408	4.762	<0.001
	Ep 5 - Ep 6	−1.475	0.418	−3.528	0.008
	Ep 5 - Ep 7	−1.702	0.413	-4.119	0.001
Whining	Ep 2 - Ep 3	−3.239	0.443	−7.314	<0.001
	Ep 2 - Ep 4	−1.388	0.463	−2.995	0.044
	Ep 2 - Ep 5	−3.485	0.446	−7.819	<0.001
	Ep 2 - Ep 6	−3.137	0.433	−7.244	<0.001
	Ep 2 - Ep 7	−1.577	0.462	−3.411	0.012
	Ep 3 - Ep 4	1.851	0.330	5.603	<0.001
	Ep 3 - Ep 7	1.662	0.319	5.213	<0.001
	Ep 4 - Ep 5	−2.098	0.334	−6.272	<0.001
	Ep 4 - Ep 6	−1.749	0.318	−5.509	<0.001
	Ep 5 - Ep 7	1.908	0.317	6.019	<0.001
	Ep 6 - Ep 7	1.560	0.301	5.180	<0.001
Head-turning	Ep 1 - Ep 2	−0.862	0.144	−5.967	<0.001
	Ep 1 - Ep 3	−0.632	0.146	−4.338	<0.001
	Ep 1 - Ep 4	−0.740	0.144	−5.153	<0.001
	Ep 1 - Ep 6	−0.962	0.141	−6.830	<0.001
	Ep 1 - Ep 7	−0.564	0.148	−3.799	0.003
	Ep 6 - Ep 7	0.398	0.120	3.315	0.016
	Anxious Ep 1 - Anxious Ep 2	−0.771	0.236	−3.267	0.019
	Anxious Ep 2 - Anxious Ep 7	0.931	0.247	3.774	0.003
	Anxious Ep 6 - Anxious Ep 7	0.805	0.250	3.217	0.022
	Avoidant Ep 1 - Avoidant Ep 3	−0.954	0.320	−2.982	0.045
	Avoidant Ep 1 - Avoidant Ep 4	−1.219	0.310	−3.930	0.002
	Avoidant Ep 1 - Avoidant Ep 6	−1.094	0.314	−3.481	0.009
	Avoidant Ep 1 - Avoidant Ep 7	−1.193	0.311	−3.839	0.002
	Secure Ep 1 - Secure Ep 2	−1.130	0.148	−7.614	<0.001
	Secure Ep 1 - Secure Ep 3	−0.628	0.158	−3.973	0.001
	Secure Ep 1 - Secure Ep 4	−0.758	0.155	−4.880	<0.001
	Secure Ep 1 - Secure Ep 6	−1.146	0.148	−7.743	<0.001
	Secure Ep 1 - Secure Ep 7	−0.659	0.157	−4.183	0.001
	Secure Ep 2 - Secure Ep 3	0.501	0.123	4.072	0.001
	Secure Ep 2 - Secure Ep 4	0.372	0.119	3.115	0.030
	Secure Ep 2 - Secure Ep 7	0.471	0.122	3.854	0.002
	Secure Ep 3 - Secure Ep 6	−0.518	0.122	−4.227	0.001
	Secure Ep 4 - Secure Ep 6	−0.388	0.119	−3.266	0.019
	Secure Ep 6 - Secure Ep 7	0.487	0.122	4.009	0.001
	Anxious Ep 2—Avoidant Ep 2	0.828	0.342	2.424	0.041
Sniffing the door	Ep 1 - Ep 3	−1.582	0.295	−5.370	<0.001
	Ep 1 - Ep 5	−1.044	0.311	−3.359	0.014
	Ep 2 - Ep 3	−2.440	0.441	−5.539	<0.001
	Ep 2 - Ep 5	−1.903	0.452	−4.213	0.001
	Ep 2 - Ep 6	−1.515	0.462	−3.279	0.018
	Ep 3 - Ep 4	1.789	0.316	5.664	<0.001
	Ep 3 - Ep 6	0.925	0.258	3.582	0.006
	Ep 4 - Ep 5	−1.252	0.332	−3.776	0.003
Locomotion	Ep 1 - Ep 2	−1.692	0.342	−4.944	<0.001
	Ep 1 - Ep 3	−1.497	0.356	−4.207	0.001
	Ep 1 - Ep 4	−2.082	0.334	−6.242	<0.001
	Ep 1 - Ep 5	−1.271	0.360	−3.533	0.008
	Ep 1 - Ep 6	−1.961	0.333	−5.886	<0.001
	Ep 1 - Ep 7	−1.723	0.341	−5.057	<0.001
	Ep 4 - Ep 5	0.811	0.221	3.672	0.005
	Ep 5 - Ep 6	−0.691	0.219	−3.153	0.027

### Correlations between behaviours and physiological variables across attachment styles

In securely attached dogs, lip-licking was weakly associated with decreased cortisol (*ρ* = −0.3583, *p* = 0.027) and increased oxytocin (*ρ* = 0.3563, *p* = 0.049), while barking was moderately negatively related to oxytocin (*ρ* = −0.4652, *p* = 0.008).

In anxious dogs, lip-licking and whining were both strongly negatively correlated with oxytocin (*ρ* = −0.7167, *p* = 0.037; *ρ* = −0.7289, *p* = 0.026), whereas locomotion showed a strong positive association (*ρ* = 0.7448, *p* = 0.021).

In avoidant dogs, door-sniffing and head-turning were positively correlated with cortisol (*ρ* = 0.7448, *p* = 0.021) and rectal temperature (*ρ* = 0.7625, *p* = 0.011), respectively.

**Figure 3.**
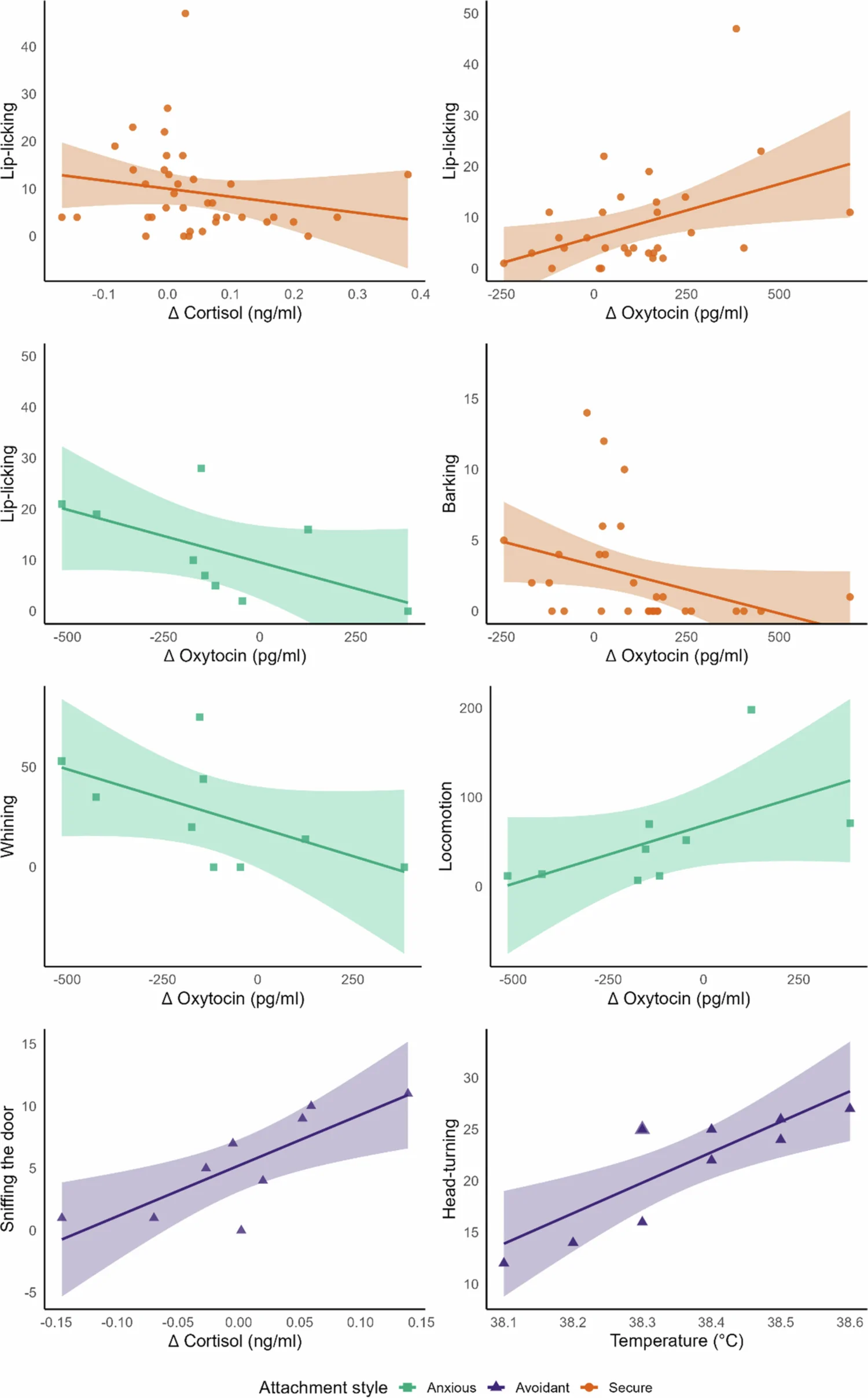
All the significant correlations between physiological parameters and behaviours across attachment styles. All behaviours were recorded as frequencies, except ‘locomotion’, which was recorded as duration (seconds).

Significant correlations are summarised in Table 6 and Figure 3.

**Table 6.** Statistically significant correlations between behaviours and physiological variables across attachment styles.

Attachment Style	Behaviour	Physiological Variable	ρ	p	CI Lower	CI Upper
Secure	Lip-licking	Δ Salivary Cortisol	−0.3583	0.027	−0.602	−0.049
Secure	Lip-licking	Δ Salivary Oxytocin	0.3563	0.049	−0.002	0.625
Secure	Barking	Δ Salivary Oxytocin	−0.4652	0.008	−0.696	−0.172
Anxious	Lip-licking	Δ Salivary Oxytocin	−0.7167	0.037	−1.000	−0.138
Anxious	Whining	Δ Salivary Oxytocin	−0.7289	0.026	−0.953	−0.200
Anxious	Locomotion	Δ Salivary Oxytocin	0.7448	0.021	0.179	0.983
Avoidant	Door sniffing	Δ Salivary Cortisol	0.7448	0.021	0.160	0.943
Avoidant	Head-turning	Rectal Temperature	0.7625	0.011	0.119	1.000

## Discussion

In this study, we used the attachment framework to explore the contextual and hormonal correlates of behavioural signals commonly used as stress indicators in dogs and we hypothesised that these behaviours would not be expressed across the episodes of the SSP at a rate suggestive of a linear relationship with the dogs’ stress levels.

As hypothesised, the expression of all the examined behaviours, such as lip-licking, shaking, paw-lifting, head-turning, door-sniffing, whining, and locomotion, differed significantly across episodes. Furthermore, these variations did not always follow the direction expected if such behaviours were linearly and positively associated with stress levels. This supports previous suggestions that these behaviours are context-dependent in their expression and function, calling for a conceptual reconsideration of how they are used and interpreted in the assessment of canine stress. By distinguishing the phenotypic expression of a behavioural signal from the meaning that the behaviour assumes as a function of context, the Message and Meaning framework appears a valid model to overcome the weaknesses of traditional paradigms that assume a direct correspondence between behaviours and stress, or, more broadly, that attribute specific cognitive or emotional states to particular signals, such as ‘appeasement signals’ or ‘displacement behaviours’. We believe that this perspective may provide us with a more suitable conceptual background for interpreting our findings and possibly understanding canine behaviour. First, we argue that these behaviours should not be aggregated into a single ‘stress behaviour’ category, as sometimes done in previous studies (Riggio et al. 2021; Hunt et al. 2022; Helsly et al. 2022; Romaniuk et al. 2022), since different behaviours may be expressed at different rates depending on the specific experimental context. Second, they should not be interpreted as linearly related to stress intensity without accounting for the broader social and environmental circumstances in which they occur.

For instance, dogs in our study performed body shaking at a significantly higher rate during the second reunion episode with the owner (episode 7), compared to the first two episodes of the procedure (episode 1 in the presence of the owner only, and episode 2 in the presence of the owner and the stranger). Although not statistically significant and therefore potentially due to chance, we believe it is worth noting that a visible increase in shaking frequency was also observed during the first reunion with the owner (episode 4). The highest frequency of shaking occurred in the second reunion (episode 7), toward the final stages of the test, suggesting that, within the context of the SSP, this behaviour may be more likely to emerge when stress levels may have accumulated, in line with progressive structure of the test. This pattern is clearer in anxiously attached individuals who, by definition, tend to display higher levels of stress during the SSP. Moreover, the increased expression of shaking specifically during reunion episodes with the owner suggests that this behaviour is more likely to occur after the acute stressor has passed, and particularly in the presence of a familiar figure. If familiarity were not a relevant factor, we would expect a similar or even higher frequency of shaking in the reunion with the stranger (episode 6) compared to the second reunion with the owner (episode 7). This interpretation aligns with the well-supported hypothesis that familiarity contributes to the caregiver’s capacity to act as a source of emotional support during stressful situations (Cimarelli et al. 2021; Buttner et al. 2023). Considering the context of the behaviour, this observation is consistent with earlier interpretations of body shaking as a behavioural expression of emotional relief (Beerda et al. 2000) or as a marker of a shift in arousal state (Rehn and Keeling 2011). Notably, Rehn et al. (Rehn and Keeling 2011) reported similar findings, in which dogs that had been left alone for longer durations, and were therefore supposed to be more stressed, displayed more shaking behaviour upon reunion with the owner. When interpreted within the Message and Meaning model, our findings also seem to align with the more recent hypothesis that shaking may serve as a marker of behavioural transition (Bryce et al. 2024), as shifts in affective or arousal states may increase the likelihood of such transitions compared to more stable conditions. This framework allows us to account for the influence of emotion or arousal on the expression of shaking while avoiding the assumption that the it carries a single, cross-contextual meaning.

Previous authors usually describe paw-lifting as an indicator of acute and chronic stress (Beerda et al., 1998; Beerda et al. 1999), as a displacement behaviour or as reflecting uncertainty (Pedretti et al. 2023). In the present study, paw-lifting was expressed significantly more in all social conditions compared to the episode in which the dog was left alone in the room (episode 5). This finding alone challenges previous interpretations suggesting that paw-lifting is a cross-contextual indicator of uncertainty. Furthermore, within the social episodes, paw-lifting was observed more frequently during the first and second reunion with the caregiver than during the episode in which the dog was separated from the owner and left with a stranger for the first time (episode 3). This pattern is difficult to reconcile with the assumption that paw-lifting is a direct indicator of stress. If the presence of the attachment figure functions as a source of emotional support—as consistently reported in the literature (e.g. Gácsi et al., 2013)—then we would have expected lower levels of paw-lifting in reunion episodes, under the assumption that it reflects stress or uncertainty. Furthermore, according to the literature, paw lifting seems to be displayed in interactions that are accompanied by different emotional states. For instance, Handelman (Handelman 2008) proposed that paw lifting may function as a metasignal indicating that either playful or agonistic interactions could follow. Additionally, paw lifting has been described as an appeasement signal in both wolves and dogs (Rugaas 2006; Mariti et al. 2017). While our study design does not allow for definitive conclusions regarding the function of paw lifting, we did observe dogs displaying this behaviour during olfactory exploration of objects within the experimental room. Therefore, if paw lifting does indeed signal anticipation of possible action, it is unlikely to be specific to agonistic encounters. Rather, it may be more broadly related to anticipation or preparation for a motor response, not necessarily driven by a single motivation or associated to a single emotional state. However, this explanation remains speculative, as the pattern of paw-lifting behaviour observed in our study does not provide sufficient information about the message (in the sense intended in the Message and Meaning model) intrinsic to this signal. Therefore, we can only suggest that paw lifting should not be used as a marker of stress, since ‘stress’ may represent just one of the many internal states that may accompany this behaviour depending on the context in which it is expressed.

As reported in the methods, head-turning frequency was not measured during the episode in which the dog was alone in the room, as we chose to record only those instances in which head-turning occurred in response to a social stimulus (i.e. any visual engagement involving the person, independent of reciprocity or which individual initiated the interaction). This decision was made to ensure that the behaviour could be reliably coded from a clearly identifiable trigger (i.e. the person). Notably, head-turning was the only behaviour recorded whose expression was significantly influenced by episode, attachment style, and the interaction between the two. Regardless of attachment style, dogs displayed higher levels of head-turning in all episodes compared to the first. This may be explained by the different levels of social engagement with the person tipically shown by dogs across episodes. In episode 1, dogs are generally more focused on exploring the novel environment of the experimental room, which may limit their responsiveness to social cues. In support of this interpretation, Pastore et al. (2011) observed that agility dogs tended to display head-turning immediately before the handler gave the starting signal, or when the handler addressed a dog that had failed to complete an obstacle during the agility course. It is therefore unclear why head-turning is often referred to as a displacement behaviour (Pastore et al. 2011), as these are typically defined as actions performed out of context or seemingly irrelevant to the immediate situation. In contrast, head-turning appears to be a highly relevant behaviour in social contexts, potentially functioning as a communicative signal of disengagement from an overwhelming or unpleasant interaction (Travain et al. 2015; Mariti et al. 2017; Pedretti et al. 2023). The observed differences in head-turning expression among dogs with distinct attachment styles provide further support for the view that this behaviour may signal disengagement (message). Although the trend of head turning observed during the test challenges its use as a reliable indicator of stress, a possible interpretation of the underlying internal state (meaning) within the context of our study protocol can only be inferred from its relationship with contextual variables, such as attachment styles and physiological measures. The trends observed for secure and anxious dogs are similar, with both groups displaying a significantly higher frequency of head-turning in the presence of the stranger (episode 6) compared to the second reunion with the owner (episode 7). This pattern is not observed in the avoidant group, which is the only group that shows a visible, although not statistically significant, increase in head-turning in the presence of the owner (episodes 4 and 7) compared to episodes involving the stranger (episodes 3 and 6). Furthermore, avoidant individuals were the only group in which head-turning was associated with a physiological variable, specifically rectal temperature. Increased body temperature is widely regarded as an indicator of sympathetic activation and acute stress (Gomart et al. 2014; Travain et al. 2015; Bragg et al. 2015). These findings are consistent with the attachment framework, which posits that avoidantly attached individuals tend to exhibit their emotional discomfort in engaging with their caregiver through higher levels of avoidance (Ainsworth et al. 2015; Riggio et al. 2021). Interpreted through the Message and Meaning framework, the observed change in body temperature in this group may reflect autonomic arousal contributing to the context-dependent construction of meaning.

Results for the door-sniffing behaviour reveal a quite clear pattern, with higher frequencies observed during episodes in which the dog was separated from the owner. The inclusion of door-sniffing amongst the behaviours assessed in our study is due to the design of the SSP (in which the owner exits the room from the same door in two different episodes leaving the dog inside), and should not be conflated with the concept of sniffing to disengage from direct social interactions, as described in previous literature (Mariti et al. 2017; Townsend and Gee 2021; Pedretti et al. 2023). Although door sniffing was expressed most frequently during episodes expected to elicit the strongest stress response in dogs (i.e. absence of the owner), most likely triggered by the owner’s exit, some instances of door sniffing also occurred when only the owner or when both the owner and stranger were present. This suggests that, while mostly associated with a negative emotional state during the SSP, door sniffing may have also reflected other internal states (e.g. different motivations or intentions) depending on the episode (context) in which it was observed. The relationship with physiological measures may help elucidate the meaning of door sniffing in dogs as a function of their attachment style. Specifically, in avoidant dogs, door-sniffing behaviour was positively correlated with changes in *Δ* cortisol concentrations, potentially indicating that individuals who displayed this behaviour experienced increased metabolic demand in response to a perceived uncertain future. This correlation aligns with the typical behavioural profile of insecure-avoidant individuals, who tend to avoid overt expressions of stress, possibly as a strategy to reduce rejection by the attachment figure (Ainsworth et al. 2015; Riggio et al. 2021; Riggio 2021). Although our findings suggest that context influences the expression of dog sniffing behaviour during the SSP, this is the only behaviour that follows the expected trend of stress fluctuations across the test episodes. This is likely because this behavioural category was specifically defined with the experimental setting and its potential associated sources of stress in mind.

Locomotion was significantly higher in all episodes compared to the first, suggesting it may be associated with the dogs’ stress levels. However, a context-related effect is also evident, as locomotion was significantly higher in Episode 6 and showed a visually increasing trend in Episode 3, both of which involved the presence of the stranger. While it is possible that the stranger acted as an additional stressor for some dogs, exacerbating the effects of separation from the owner, most dogs appeared to seek proximity to and comfort from the unfamiliar person, using them as a surrogate attachment figure in the absence of their primary caregiver. Hence, a more plausible explanation is that the presence of the stranger introduced a second social target—in addition to the door—toward which the dogs directed their attention, resulting in increased movement between the two. Although locomotion was statistically higher in Episode 4 than in Episode 5, this difference does not reflect its overall visual trend across episodes. Our best explanation for this finding is that the statistical outcome has been influenced by the the high inter-individual variability observed for this behaviour in Episode 5.

In anxiously attached dogs, locomotion was positively correlated with changes in oxytocin levels. In our study, this behaviour was originally operationalised as an indicator of stress (Beerda et al. 2000), and was recorded when dogs engaged in non-focalised, non-exploratory, and non-playful movement, interpreted as potentially anxiety-driven wandering within the experimental room. Given the exploratory nature of this study, in which oxytocin was measured at only two time points (before and after the test), we cannot provide evidence of directionality in the relationship. On one hand, it may be that higher levels of locomotion in anxiously attached individuals lead to higher concentrations of oxytocin, possibly supporting the hypothesis of an overall anxiolytic effect of oxytocin in response to stress (Yoon and Kim 2020). On the other hand, the findings may align with the idea that oxytocin enhances sensitivity to socially relevant cues (Guastella et al. 2008; Ross and Young 2009), such that anxiously attached individuals, who tend to perceive their caregiver as inconsistently available or responsive, may experience increased, rather than reduced stress when oxytocin levels are elevated. However, our interpretations remain speculative, and warrant further research to clarify the relationship between oxytocin and stress response in dogs with different attachment styles.

Regardless of the dog’s attachment style, lip-licking was expressed significantly less in Episode 1 than in any other episode. This may suggest that lip-licking increased as the procedure progressed, potentially reflecting internal stress levels. However, if lip-licking were directly and exclusively linked to stress, we would expect it to occur more frequently during episodes involving the owner’s absence, compared to those in which a person is present. This is not the case, as lip-licking was displayed significantly more during both the first reunion with the owner (Episode 4) and the reunion with the stranger (Episode 6) than during the episode in which the dog was left alone (Episode 5). These findings suggest that the expression of lip-licking is context-dependent, with a higher likelihood of occurrence in the presence of a social stimulus. It is important to note that Episode 1 is not only the episode in which stress levels are presumed to be lowest, but also the only episode in which a social stressor has not yet been introduced, which explains why stress levels are still low. This is the reason why, at this early stage of the procedure, most dogs appear to direct their attention toward the novel environment rather than the human figure (Prato-Previde et al. 2003). Similarly, our findings do not support the hypothesis that lip-licking functions primarily as an appeasement behaviour. If this were the case, we would expect lip-licking to occur more frequently in the presence of an uncertain social stimulus, such as the stranger, compared to the caregiver. However, not only we found no statistically significant difference in this regard, but a trend for increased lip-licking in the presence of the stranger was observed only in anxiously attached individuals. In contrast, secure and avoidant individuals displayed more lip-licking in the presence of the caregiver. Furthermore, previous studies have reported the expression of lip-licking during affiliative interactions, such as the reunion with the owner (Rehn and Keeling 2011), and during positive anticipation (Caeiro et al. 2017). These findings suggest that lip-licking is not exclusively associated with frustration or negative emotional states (Firnkes et al. 2017; Albuquerque et al. 2018; Bremhorst et al. 2019, 2022). It is also important to emphasise that a higher frequency of a behaviour in a given context does not imply that the behaviour is exclusive to that context. For instance, Firnkes et al. (2017) found that during friendly greetings with a human stranger, 59% of dogs displaying a submissive approach showed lip-licking, compared to 32% of dogs showing a sociopositive approach. Based on this, the authors suggested that lip-licking may function as an appeasement signal. However, they did not discuss the possible function of lip-licking in dogs that displayed confident, sociopositive approaches without signs of social uncertainty, leaving open the question of whether lip-licking may serve multiple communicative or emotional functions depending on context and individual differences. Similarly, some dogs in our study performed lip-licking even when they were alone in the room. Individual instances of behavioural expression that deviate from normative trends should also be considered when attempting to formulate cross-contextual explanations of behaviours. Taken together, our findings suggest the following: 1) No specific emotional valence appears to be uniquely associated with lip-licking, as the behaviour was observed in every episode of the SSP; 2) In social contexts, such as the SSP, lip-licking may occur even in the absence of a social stimulus, although it is more likely to be expressed when a social stimulus is present (i.e. Episodes 4 and 6 more than Episode 5); 3) Lip-licking may occur more frequently in conditions in which the dog‘s attentional focus is on the social interaction, with higher expression across all episodes involving social components (including Episode 5) compared to the baseline (Episode 1). Therefore, some may argue that lip-licking may function as indicator of social arousal, rather than a clear marker of either positive or negative emotional valence. However, Caeiro et al. (2017) found that lip-licking tends to be replaced by other behaviours, such as overt aggression or avoidance, as the perceived threat from a social stimulus increases, along with arousal levels. Again, we propose that the application of the Message and Meaning model may provide a more suitable interpretative framework to disentangle the cross-contextual features of lip licking from its context-dependent meanings. Based on this theoretical framework, a possible interpretation of our findings is that lip-licking may convey the message of perceptual engagement with external stimuli, rather than a reaction to threat per se. This may explain why in scientific literature it is reported to be performed in association with qualitatively different emotional states, but more frequently during negative interactions (Firnkes et al. 2017; Albuquerque et al. 2018; Bremhorst et al. 2019, 2022). Evolutionary speaking, ignoring social cues with positive valence may have less grave consequences than ignoring social cues that may represent a threat. The negative association between lip-licking and *Δ* salivary cortisol in secure dogs, as well as the opposite relationships with salivary oxytocin in secure and anxious dogs, may help elucidate the meaning of this behaviour across different attachment styles. Although limited by the exploratory nature of our approach, an explanation consistent with the attachment paradigm and the reported cognitive effects of oxytocin could be that in securely attached dogs, who tend to perceive their caregiver as consistently responsive and supportive in times of distress, higher levels of oxytocin may enhance the salience of social stimuli which, being perceived as positive, in turn increase the level of social engagement. Conversely, in anxiously attached dogs, the same oxytocin-driven increase in sensitivity to socially relevant cues may actually reduce social engagement, as cues from an inconsistently supportive caregiver may trigger feelings of uncertainty and unpredictability.

A similar mechanism, may explain our findings on the negative association between vocalisations (barking in secure individuals and whining in anxious individuals) and oxytocin. Specifically, higher oxytocin concentrations in securely attached individuals may enhance the positive effects of socially relevant cues from available and sensitive caregivers, thereby increasing trust and reducing behaviours aimed at regaining proximity, such as barking (Lund and Jørgensen 1999; Pongrácz et al. 2017). In contrast, in anxiously attached dogs, the oxytocin-induced sensitivity to social cues may amplify the negative emotional valence of these cues and reduce the dog’s attempts to regain proximity to an inconsistent and unpredictable caregiver, thereby leading to a decrease in whining (Pongrácz et al. 2017). A similar effect has been observed in humans, where intranasal administration of oxytocin enhanced the negative bias in the recollection of maternal care among more anxiously attached individuals (Bartz et al. 2010). This interpretation appears to be consistent with the human literature, where the social effects of oxytocin appear to be mediated by attachment style (Bartz et al. 2011; Fang et al. 2014), through the amplification of pre-existing interpersonal schemas. However, we would like to emphasise that the design of our study does not allow us to determine the direction of the association between behaviours and physiological parameters. Consequently, a reversed causal relationship - namely, that reduced vocalisations lead to higher increases in oxytocin - cannot be ruled out and may be statistically equally plausible.

One of the main limitations of this study is the relatively small sample size. Although the total number of experimental subjects exceeds that of most studies on dog–owner attachment, the high proportion of securely attached individuals results in a relatively low absolute number of anxiously and avoidantly attached subjects. While this imbalance reflects the natural distribution of attachment styles in the pet dog population, where secure attachment is typically the most frequent, it may have limited the statistical power of the GLMMs to detect more subtle differences between attachment styles. Our findings should therefore be interpreted with caution and considered as a starting point for further research with larger, more balanced cohorts. From a statistical perspective, while we acknowledge the potential carryover effect of behavioural responses from one episode to the next, we did not include previous-episode behaviour (lag-1) as a covariate. This decision was made because our primary focus was on understanding context-dependent changes in behaviour, and we felt that incorporating lag-1 might have introduced unnecessary complexity, potentially removing meaningful variability associated with the attachment style. Equally important, the SSP was not originally designed for the purposes of this study. However, due to its structured format, it may offer a valuable opportunity to explore contextual factors that may help explain the underlying function of various stress responses in dogs. That said, more targeted assessments designed to expose dogs to a variety of environmental and social contexts are needed to test our hypotheses and deepen our understanding of the significance of these behaviours. Finally, while the percentage of dogs classified as having a specific attachment style in our study falls well within the ranges reported by previous research, it must be noted that these ranges are extremely broad and vary considerably across studies (secure: 50% [8/16]–80% [32/40]; anxious: 4% [1/27]–51.6% [16/31]; avoidant: 0% [0/21]–24% [6/25]; disorganised: 0% [0/40]–20% [10/51]) (Ainsworth and Wittig 1969; Topál et al. 2005; Mongillo et al. 2013; Nagasawa et al. 2015; García-Pérez 2023). This may highlight the need for a standardised laboratory test, as well as unified classification system for attachment styles among research groups working in the field of canine attachment, which would significantly improve the scientific validity, reliability, and cross-study comparability of research on dog attachment. That said, more targeted assessments designed to expose dogs to a variety of environmental and social contexts are needed to test the hypotheses form this study and deepen our understanding of the significance of these behaviours.

More broadly, while the need for standardised terminology and validated criteria for behavioural assessment is indisputable in both clinical and non-clinical behavioural science, it is essential to acknowledge that such criteria remain human constructs. Even with improved standardisation, human-defined categories and labels may not fully capture the underlying biological reality, which may exist on a continuous spectrum of individual variation rather than as discrete units. This variation is likely driven by a complex interaction of genetic predispositions and life history, suggesting that while our current constructs are invaluable for analysis, they represent a simplified map of a much more fluid biological landscape. Notably, in the context of the present study, this epistemological perspective may apply to both attachment classification and behavioural categorisation.

## Conclusions

The findings from this study may suggest the potential value of a framework that helps disentangle the confusion created by historically classifying canine behaviours as stress-related based on their presumed emotional or functional valence. By considering a model that separates the stable, universal component of a behaviour from its context-dependent features, such as the Message and Meaning model, our observations seem to suggest that the behaviours examined in this study do not necessarily carry an intrinsic emotional valence, and that their relationship with stress may depend, for each specific behaviour, on the context in which they occur. From a practical perspective, our findings may underscore the value of evaluating each behaviour independently and within its context, in both experimental and clinical settings and avoid labelling the assume specific motivations, intentions or emotional state to be fixed for specific behaviours (i.e. displacement behaviours, appeasement signals). Despite its limitations and opportunistic nature, this study provides preliminary insights into an alternative approach to the classification and interpretation of behaviours observed in dogs during stressful situations, with potential implications for canine welfare and their integration into human society.

## Supplementary Material

Supplementary Material VQ SM 2nd.docx

## Data Availability

The dataset from this study is publicly available on Zenodo at the following link: https://doi.org/10.5281/zenodo.17416858.
