# Supplementary material for: Rethinking dog stress behaviours: contextual and physiological insights from the attachment framework
Source: Vet Q. 2026 Aug 21;46(1):2682284. doi: 10.1080/01652176.2026.2682284 (PMC13501743; doi:10.1080/01652176.2026.2682284)
Supplement: Supplementary file 1 [file TVEQ_A_2682284_SM9658.docx]

**Table S1:** Dog attachment styles and corresponding descriptions (Riggio et al., 2021)

| **Attachment Style** | **Description** |
| --- | --- |
| Secure | The dog actively seeks proximity with the caregiver (e.g., approach, physical proximity, contact and/or persistent gazing), and such proximity is increased in quality and/or intensity after separation, especially upon the second reunion.   The reunion with the caregivers (their mere presence or, for other dogs, the proximity or the contact with them) is responsible for calming the dog in case he was distressed at separation. It may take some time, but during the reunion episode the dog, if excited/distressed at the beginning of the episode itself, appears reassured by the presence of or interaction with the caregiver, so that he can either explore the environment, lay down, remain in proximity/contact with the caregiver, always in a relaxed manner.   The dog may show some interest in the stranger, thus can greet her and stay in proximity/contact with her. However, the dog shows a preference for the caregiver (e.g., more intense greeting, longer or deeper proximity seeking, reassurance).   During separation, especially when left alone in the room, the dog may show some search of the caregiver (e.g., sniffing as searching, going close to the chair of the caregiver, looking at the door, staying close to the door, scratching at the door), and may display some distress at separation (e.g., vocalizations). When separated from the caregiver but in the presence of the stranger, the dog may remain close to the stranger for reassurance.   Before separation, especially before the first separation, the dog may show interest in the environment, thus exploring the room and the stranger (for the latter, it can be displayed as sniffing, greeting). Such interest for the environment may remain even after separation, but it is usually overshadowed by the increase in proximity seeking. In certain cases, such as dogs not used to novel environments or places similar to the experimental room, exploration may be reduced. |
| Avoidant | The dog shows little or no tendency to actively seek proximity with the caregiver (e.g., approach, physical contact and/or persistent gazing), and such proximity is not increased in quality and/or intensity after separation, instead it may be reduced upon the second reunion. The dog shows some proximity avoidance: the dog may follow or approach the caregiver but the sequence is quickly interrupted, so that the dog turns, looks or moves away. The dog shows some gaze aversion, e.g., alternate gazing from the caregiver to somewhere else not clearly identified (i.e., not the door).   The dog may show some interest in the stranger, thus can greet her and stay in proximity/contact with her. The dog does not show a marked preference for the caregiver (e.g., more intense greeting, longer or deeper proximity seeking, reassurance).   During separation, the dog may display some/little search of the caregiver (e.g., sniffing as searching, going close to the chair of the caregiver, looking at the door, staying close to the door), and shows little distress at separation (e.g., vocalizations). Distress at separation may be more pronounced when the dog is left completely alone. When separated from the caregiver but in the presence of the stranger, the dog may remain close to the stranger for reassurance. Before separation, especially before the first separation, the dog may show interest in the environment, thus exploring the room and the stranger (for the latter, it can be shown as sniffing, greeting). Such interest for the environment may remain even during and after separation. In certain cases, such as dogs not used to novel environments or places similar to the experimental room, exploration may be reduced. |
| Anxious | The dog actively and obviously seeks proximity with the caregiver (e.g., physical proximity, contact and/or persistent gazing), and such proximity is increased in quality and/or intensity after separation, especially upon the second reunion. Differently from the secure style, the dog cannot find reassurance in the caregiver, so that the dog makes strong efforts to maintain physical contact with the caregiver (e.g., physically intrusive behavior) and this is combined with persistent distress (e.g., the dog can keep vocalizing, bite/chew the owner).   The dog may show interest in the stranger, thus can greet her and stay in proximity/contact with her. However, the dog shows a marked preference for the caregiver (e.g., more intense greeting, longer or deeper proximity seeking, reassurance).   During separation, especially when left alone in the room, the dog shows some search of the caregiver (e.g., sniffing as searching, going close to the chair of the caregiver, looking at the door, staying close to the door, scratching at the door), and displays evident distress at separation (e.g., frequent vocalizations, pacing). When separated from the caregiver but in the presence of the stranger, the dog may remain close to the stranger for reassurance.   Before separation, especially before the first separation, the dog may show little interest in the environment, not exploring the room nor focusing on the stranger (e.g., sniffing, greeting) for a long time. If displayed, such interest for the environment does not remain after separation. |
| Disorganised | There is not a clear pattern; behavior is inexplicable or contradictory in the context of interaction with the caregiver, and this lack of organization has to be frequent, extreme, or extensive and more evident in the caregiver’s presence than absence. Particular relevance is given to repeated manifestations of disorganization, the appearance of several different indices of disorganization, and disorganized behavior displayed immediately after reunion. |
| Unclassified | The dog behavior seems disturbed but too ambiguous to classify, and an alternative underlying condition can be supposed, either a physical illness or a behavioral disorder. For instance, the dog shows constantly repetitive behaviors regardless of the caregiver presence (possibly due to neurological or compulsive disorder), or the dog shows lethargy. |
